# Hydatidose cérébrale multiple d'origine cardiaque: à propos d'un cas

**DOI:** 10.11604/pamj.2015.22.15.7743

**Published:** 2015-09-09

**Authors:** Mamadou Bata Dianka, Tarek El Hamdani, Ibrahima Kaba, Abdessamad Naja, Khadidia Ibahioin, Abdessamad El Azhari

**Affiliations:** 1 Service de Neurochirurgie, CHU Ibn Rochd, Casablanca, Maroc

**Keywords:** Hydatidose, cérébrale, cardiaque, hydatidosis, cerebral, cardiac

## Abstract

L'hydatidose cérébrale est très rare, de bon pronostic après le traitement chirurgical. La forme multiple de l'hydatidose cérébrale rend difficile la prise en charge chirurgicale et altère le bon pronostic de cette pathologie. Nous rapportons l'observation d'une hydatidose cérébrale multiple d'origine cardiaque chez une fillette de 06 ans amenée aux urgences pour crises convulsives généralisées et un syndrome d'hypertension intracrânienne. L'examen clinique était normal, la tomodensitométrie a révélé 8 kystes hydatiques cérébraux se situant à la fois en sus et sous tentoriel et des deux côtés de la faux du cerveau. Elle fut opérée en deux temps avec une bonne amélioration clinique. Nous soulignons à travers ce cas, la rareté et la difficulté de la prise en charge chirurgicale de l'hydatidose cérébrale multiple.

## Introduction

L'hydatidose cérébrale multiple est exceptionnelle même dans les pays endémiques. Elle compliquerait le plus souvent une localisation cardiaque. C'est une affection parasitaire causée par l'echinococus granulosis. Le tableau clinique, comme dans tout processus intracrânien, est fait d'un syndrome d'hypertension intracrânien et des signes de focalisation selon la localisation. La tomodensitométrie permet le diagnostic positif. Le traitement chirurgical est complexe dans la forme multiple.

## Patient et observation

**Figure 1:**
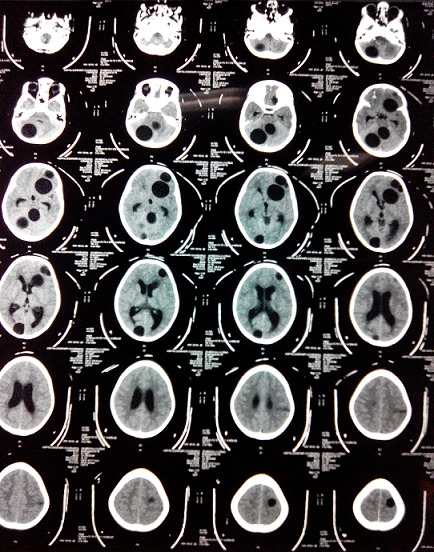
Scanner cérébral C- montrant les 8 kystes cérébraux

**Figure 2:**
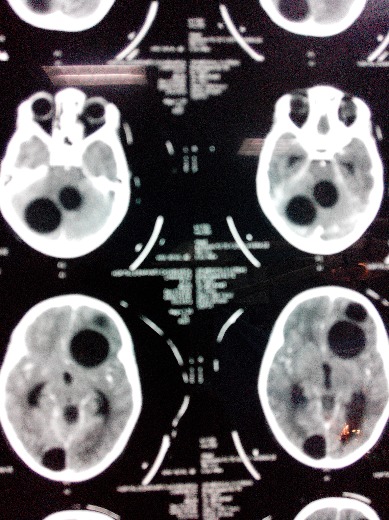
Scanner cérébral C+

**Figure 3:**
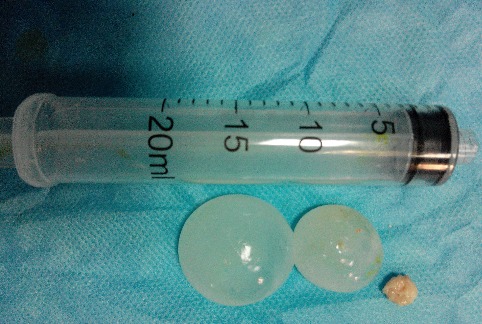
Trois Kystes cérébraux dont un déjà rompu

**Figure 4:**
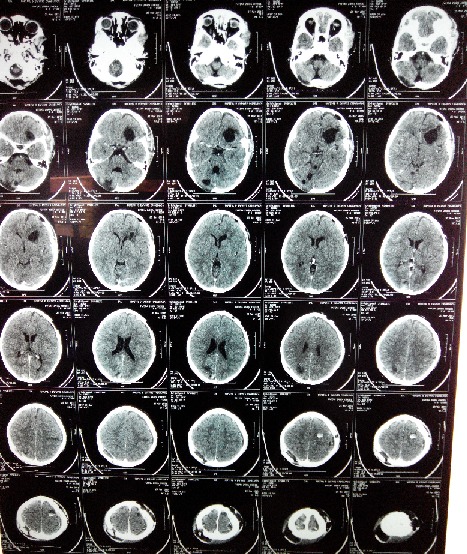
Scanner cérébral C+ après ablation totale des kystes

Une fillette de 06 ans, habitant en milieu rural avec notion de contact avec les chiens, opérée 9 mois au paravent d'un kyste hydatique cardiaque. Elle fut admise aux urgences après des crises convulsives généralisées faisant suite à des céphalées et vomissements. L'examen clinique retrouvait un enfant conscient, sans déficit neurologique. La tomodensitométrie cérébrale réalisée a montré 8 formatons kystiques (Figure 1, Figure 2), arrondies, bien limitées, sans prise de contraste en périphérie dont quatre en frontal droit difficile de déchiffrer (mesurant 18, 20, 26 et 39 mm de diamètre), un en pariétal gauche (de 20 mm), un en occipital droit (de 27 mm), enfin deux dans la fosse cérébrale postérieure mesurant 30 et 36 mm dont le plus médian comprimait le quatrième ventricule (V4) et l'aqueduc de Sylvius entrainant en amont une hydrocéphalie active. Ce bilan a été complété par une radiographie thoracique et une échographie abdominale qui sont revenues normales. La patiente fut opérée en urgence, nous abordons en premier lieu les kystes en sous tentoriel. En décubitus ventral, nous réalisons une craniectomie sous occipitale élargie à droite, une cortectomie cérébelleuse et par ponction aspiration, nous évacuons les deux kystes avec protection du parenchyme cérébelleux par du coton imbibé de sérum salé hypertonique. Dans un deuxième temps, nous retournons la patiente et abordons les kystes frontaux gauches. Nous évacuons successivement les 4 kystes par hydro pulsion (technique d'Arana Iniguez) dont deux étaient déjà rompus (Figure 3). Nous décidons de ne pas opérer dans l'immédiat les deux autres kystes restant (en pariétal gauche et en occipital droit). Au réveil, la patiente a rejoint sa place au service de neurochirurgie. Après 6 jours d'hospitalisation et sous Albendazole à la dose de 10 mg/kg/jour, la patiente est sortie de l'hôpital après disparition totale du syndrome d'hypertension intra crânienne. L'enfant fut réhospitalisée 1 mois après sa sortie pour évacuer les deux kystes restants. Au cours de cette dernière opération, nous évacuons en 1ier lieu le kyste en pariétale gauche par hydro pulsion alors que celui en occipital droit a été évacué par ponction aspiration. La patiente est sortie du service en bon état général et sans déficit neurologique avec un contrôle scannographique montrant l'ablation totale des huit kystes (Figure 4).

## Discussion

L'hydatidose se localise dans 1 à 2% des cas au niveau cérébral [1–5]. Cette rareté s'explique par l'existence de deux filtres hépatique et pulmonaire qui empêchent l'arrivée du parasite au niveau cérébral [6]. Le foie et les poumons sont alors les localisations les plus fréquentes (respectivement 60% et 30%) [4]. Elle se rencontre plus fréquemment chez l'enfant et l'adulte jeune (50 à 70%) [3, 4, 6, 7]. L'hydatidose cérébrale multiple est exceptionnelle et serait secondaire dans la majorité des cas à une localisation cardiaque [1, 6, 8]. La localisation sus tentorielle serait plus fréquente et quelques rares cas de localisation intra ventriculaire ont été décrits [4, 6, 7, 9]. L'hypertension intracrânienne est quasi constante, elle exprime la présence d'un processus expansif intracrânien qui s'installe de façon progressive sur plusieurs semaines voir plusieurs mois, du fait de la croissance lente du kyste et de l'élasticité relative de la boite crânienne de l'enfant. Les troubles visuels sont difficiles parfois à mettre en évidence chez l'enfant jeune mais l'œdème papillaire uni ou bilatérale sont souvent retrouvés lors de l'examen ophtalmologique [5]. Les cas de révélation par un coma ont été décrits [3]. La TDM cérébrale est l'examen de choix pour faire le diagnostic, elle permet d'objectiver la lésion, de préciser le nombre, le contenu, le siège et surtout les caractéristiques: Image kystique hypodense, en plein parenchyme de même densité que le LCR, à paroi fine, refoulant le parenchyme et la ligne médiane, sans prise de contraste ni d'œdème perilésionnel [5–7]. L'IRM est plus précise, elle montre le kyste hydatique cérébral sous forme d'une image liquidienne a paroi fine, avec les mêmes caractéristiques que le LCR: hypo intense en T1 et hyper intense en T2, l'absence de prise de contraste en périphérie est plus nette, et les rapports avec les structures avoisinantes sont mieux étudiés. L'IRM permet également une meilleure étude des kystes hydatiques multiples, des cas atypiques ou compliqués [1, 2]. Le bilan biologique est non spécifique et la sérologie hydatique est souvent négative [1, 7]. Une radigraphie thoracique et une échographie abdominale s'imposent à la recherche d'autres localisations associées (dans 10% des cas) [3]. Le traitement est chirurgical et l'idéal est de retirer ces kystes intacts [10]. La technique d'évacuation du kyste par hydro pulsion est la plus utilisée (décrite par Arana Iniguez). La ponction aspiration du kyste est réservée aux localisations profondes et ou étroites comme le tronc cérébral ou la fosse cérébrale postérieure de façon générale [4, 5, 10]. Le traitement médical à base d'albendazol est indiqué en cas de rupture, ou en cas de localisations multiples [5].

## Conclusion

Le kyste hydatique cérébral est une affection rare. Il existe encore malheureusement des zones endémiques et touche principalement les enfants. Le tableau clinique est dominé par le syndrome d'hypertension intracrânien. La tomodensitométrie est l'examen de base pour le diagnostic positif. La recherche d'autres localisations doit être systématique. La chirurgie est le traitement idéal, celle-ci est souvent difficile dans la forme de localisation cérébrale multiple qui peu assombrir le bon pronostic de cette affection.
